# Quantifying aerosol and microbial exposure of healthcare workers in endoscopy suites: a time-based study

**DOI:** 10.3389/fpubh.2025.1634327

**Published:** 2025-08-29

**Authors:** Meng Dan Ye, Ning Ning Li, Wen Li, Qi Han Wu, Ying Ying Wang, Bing Ru Li, Yuan Sheng

**Affiliations:** ^1^School of Nursing, Nanjing University of Chinese Medicine, Nanjing, China; ^2^Department of Gastroenterology, Nanjing Drum Tower Hospital Clinical College of Nanjing University of Chinese Medicine, Nanjing, China; ^3^Department of Gastroenterology, Nanjing Drum Tower Hospital, Affiliated Hospital of Medical School, Nanjing University, Nanjing, China

**Keywords:** endoscopy, healthcare workers, aerosols, microorganisms, qualified rates

## Abstract

**Objectives:**

This study aimed to quantify aerosol and microbial exposure levels during different working hours, analyze temporal air pollution trends in the endoscopy suite, and provide evidence to optimize infection prevention strategies.

**Methods:**

A portable laser particle counter and an airborne bacteria sampler were used to measure aerosol particle concentrations and microbial loads at four time points: before the commencement of work (baseline), and 1, 2, and 3 h after work initiation. Continuous data collection was conducted over 10 consecutive working days. Air quality assessments were performed through scientific evaluation according to relevant international and national standards.

**Results:**

Qualified rates for aerosols ≥0.5 μm increased by 20–30% during the 3 working hours compared to baseline levels. Microbial qualified rates remained at 100% throughout the study period. Aerosol concentrations across all particle sizes significantly increased with working hours (*p <* 0.01). The growth rate of 5–10 μm aerosols was higher than that of 0.3–5 μm particles (*p <* 0.01). After 3 h, concentrations of 0.5–25 μm and 0.3–10 μm aerosols increased by 1.48-fold and 1.3-fold compared to baseline values. Total microbial colonies positively correlated with work duration, polyp detection frequency, biopsy procedures performed, and procedure duration (*p <* 0.05). Microbial analysis identified 12 species, predominantly bacteria (79.6%) and fungi (20.4%).

**Conclusion:**

This study highlights trends in aerosol and microbial contamination over time and identifies four factors influencing microbial counts in the endoscopy suite. We propose some recommendations to reduce exposure risks for HCWs and patients.

## 1 Introduction

Esophagogastroduodenoscopy and colonoscopy are important tools to detect and diagnose lesions in the esophagus, stomach, and colon. Through regular screening and diagnosis, the morbidity and mortality of digestive system cancers can be effectively reduced (1–3). A total of 29.37 million esophagogastroduodenoscopies were performed in China in 2019, up 32.0% from 2012, according to the results of the 2020 China Census of Gastrointestinal Endoscopy Technology (4). Data from another national survey showed that the average annual volume of endoscopies in 2019 was 1.45 times higher than that of 2015, which means that the number of gastroenteroscopies in China will show a continuous upward trend (5). Over 2 million gastrointestinal endoscopies were performed in the UK in 2019, including around 900,000 upper GI endoscopies and 700,000 colonoscopies, and the total number of endoscopies has been increasing over the last 5 years (6, 7). In addition, both Korea and Japan have introduced nationwide screening with upper endoscopy or upper gastrointestinal series (UGIS) every 2 years (8, 9). Generally, the number of endoscopies globally is expected to continue its growth trend in the future with the aging population, promotion of cancer screening programs and advancement in endoscopy technology.

Studies have shown that esophagogastroduodenoscopy and colonoscopy are both aerosol-generating procedures (AGPs) whose diagnostic and therapeutic procedures promote the release of aerosols containing pathogenic microorganisms (e.g., bacteria, fungi, viruses) into the environment (10–12). Specifically, these microbial aerosols can be broadly classified according to particle size as follows: Bacterial cells and spores range from 0.3 to 10 μm in diameter; fungal spores range from 0.5 to 30 μm; and viruses range from 0.02 to 0.30 μm in diameter (13). During upper gastrointestinal endoscopy, patients may cough, vomit, and other physiologic responses, generating aerosols that carry microorganisms from the oral cavity, pharynx, and upper gastrointestinal tract, while during colonoscopy, patient bowel evacuation or fecal splattering may release aerosols containing intestinal microorganisms (14–16). If patients are infected or colonized with respiratory pathogens, there is an increased risk of infection among Healthcare Workers (HCWs) during endoscopic diagnostic and therapeutic procedures, and COVID-19 is transmitted in this way (17, 18). These microbial aerosols suspended in the air may induce adverse reactions through oxidative stress and inflammation when inhaled (19). Aerosols smaller than 5 μm may be inhaled into the lower respiratory tract or even directly infiltrate the alveoli, which could lead to the development of a variety of lung diseases, while aerosols in the 5–10 μm particle size range are usually deposited in the upper respiratory tract, which can increase the risk of diseases such as tooth decay and gum infections (20). Faridi et al. (21) found that exposure to bacterial and fungal aerosols could cause systemic inflammation and even cardiovascular disease. Linares et al. (22) revealed that inhalation of microbial aerosols increased the risk of dementia. Studies have shown that aerosol and microbial contamination not only increases the risk of occupational infection among HCWs, but also may threaten patient health and lead to problems such as cross-infection (23, 24). The confined space of the endoscopy suite, where aerosols tend to accumulate and are difficult to quickly diffuse and dilute, combined with long working hours and an increasing volume of consultations year after year, places HCWs at a significantly increased risk of exposure to microbial aerosol contamination (25).

Currently, most studies on the potential infection risk of aerosols to HCWs are based on case reports and specific events rather than quantitative analysis through ambient air sampling (14, 24, 26). Moreover, most of the studies failed to analyze aerosol and microbial loads under internationally or nationally recognized air quality standards and could not accurately assess air pollution levels in real clinical environments. This study aimed to quantify the aerosol and microbial exposure levels of HCWs in endoscopy suites under different working hours through prospective environmental monitoring, to reveal the temporal pattern of change of air pollution, to explore the factors affecting the exposure risk, and to provide scientific basis for the development of effective infection prevention and control strategies.

## 2 Materials and methods

### 2.1 Characteristics of the endoscopy suite

The endoscopy suite was selected randomly from the gastrointestinal endoscopy center of a tertiary hospital in Nanjing, which handles an annual patient volume exceeding 150,000. The room has a total area of 20 m^2^ with a ceiling height of 2.5 m. The indoor temperature was maintained at 24–26°C, and relative humidity was controlled between 40 and 50%. The room is relatively enclosed, featuring standard sliding doors and an air exchange vent at the entrance. Additionally, it is equipped with a fresh air system that provides a minimum of 12 air changes per hour (ACH) and 100% fresh air. The system utilizes a primary G4 filter (filtration efficiency >90% for particles ≥5 μm); however, it does not meet the standards of High Efficiency Particulate Air (HEPA) filters. Qualified technicians perform routine system maintenance, including monthly cleaning and quarterly filter replacement. All endoscopic procedures (transoral gastroscopy and colonoscopy) were performed under mild-to-moderate sedation, with patient recovery occurring in a designated area. The endoscopy suite was staffed with one endoscopist (board-certified as an attending physician, with over 5 years of experience in gastrointestinal endoscopy), one specialized endoscopy nurse, and one anesthesiologist (both with over 3 years of experience). The endoscopic equipment utilized in procedures was manufactured by Olympus Corporation.

### 2.2 Experimental design

From January 13 to February 26, 2025, the indoor air in the endoscopy suite was analyzed. Two postgraduate researchers from the hospital collected aerosol and microbial data during morning time slots over 10 consecutive workdays. Baseline measurements were conducted after the endoscopy suite had been inactive for over 12 h, with baseline data recorded as 0 h. HCWs initiated preparatory work at 8:00 a.m. daily. Timing began when the first patient entered the room, with aerosol concentrations and microbial counts measured hourly (designated as 1 h, 2 h, and 3 h, respectively). Due to the disruption caused by a 30-min lunch break, which interrupted the continuous monitoring, data collection was limited to three consecutive morning sessions. A retrospective data collection was conducted from three distinct periods, including the number of gastroscopy and colonoscopy procedures performed, procedure duration (defined as the time interval from endoscopic insertion to complete withdrawal), and the number of adenomas detected, for subsequent analysis of influencing factors.

**Figure 1:**
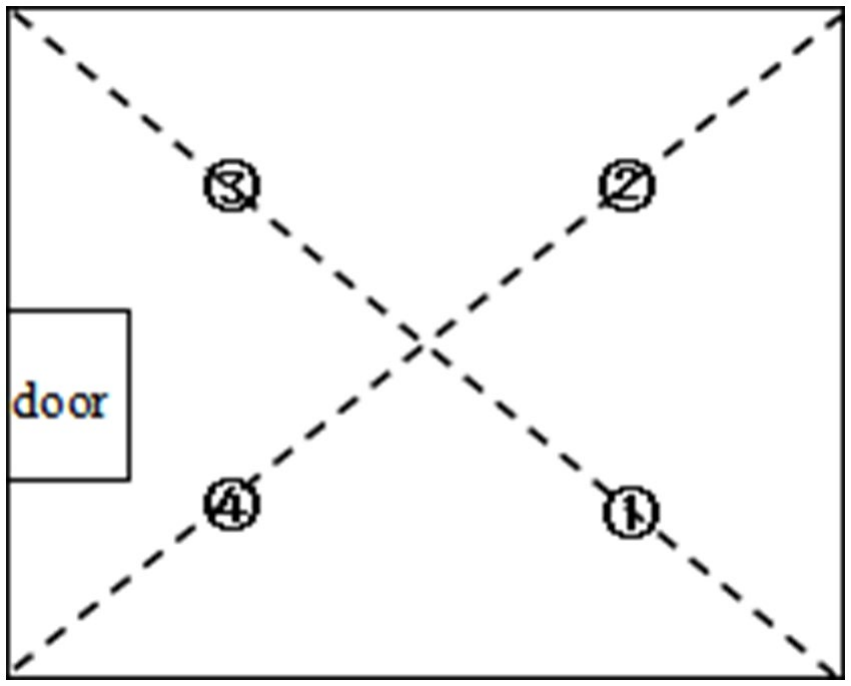
Schematic diagram of sampling points.

Four sampling points (①, ②, ③, ④) were placed at the midpoints of the room’s diagonals according to national standards (27) (Figure 1). The mean values from all four sampling points were used to represent aerosol and microbial concentrations in the endoscopy suite. The first researcher conducted aerosol sampling using the particle counter, initiating at point ① and progressing sequentially to points ②, ③, and ④. At each sampling location, three consecutive 1-min measurements were recorded. The second researcher performed microbial sampling with the airborne microbial sampler, starting at point ③ and following the sequence ③ → ④ → ① → ②, with one 5-min sampling cycle per point. A blank agar plate was included in each sampling session as a control. Following sampling, plates were promptly stored in a constant-temperature incubator at 2–8°C and subsequently transported to the laboratory for a 48-h incubation period at 35–37°C. Both the particle counter and the microbial sampler were maintained at a consistent vertical height of 0.8 m above the floor throughout the sampling process, in compliance with the environmental monitoring requirements of ISO 14644-1:2015, GB 50333–2013, and DB11/T 408–2016 (27). The door remained closed except for patient entry/exit and endoscope transfers. Three HCWs and two researchers strictly wore disposable surgical caps, surgical masks, gloves, surgical gowns, isolation suits, and surgical shoes. Both researchers rigorously followed aseptic protocols during the sampling procedures.

### 2.3 Experimental methods

Aerosol measurements were performed using a portable laser particle counter (Suxin, model SX-L310A, China) based on light scattering principles. Airborne particles were irradiated with a laser beam, and their size distribution (0.3–25 μm) and concentration were quantified by real-time analysis of scattered light intensity. The sample flow rate is 28.3 L/min ± 5% with 6 particle size channels: ≥0.3, ≥0.5, ≥1, ≥5, ≥10, ≥25 μm. Particle diameters are denoted as Dp (e.g., Dp ≥ 5 μm denotes particles with diameters ≥5 μm; Dp5-10 μm represents the 5–10 μm fraction calculated as Dp ≥ 5 μm minus Dp ≥ 10 μm). The surfaces of the instrument, sampling racks, hoses, and isokinetic sampling heads were disinfected with 75% ethanol before and after each sampling session. Calibration validity was confirmed by three consecutive zero-count measurements within a 10-min pre-sampling interval.

Airborne microbial sample was performed using an inertial impaction-based bioaerosol sampler (Suxin, model SX-JCQ-6, China). This device aspirates airborne microorganisms through a multi-orifice sampling head (flow rate: 100 L/min ± 5%) and deposits them on φ90 mm × 15 mm nutrient agar plates (Bosai Biotechnology Co., Ltd., Zhengzhou, China) containing dehydrated agar powder. After sampling, the plates were sealed and transported under temperature-controlled conditions (2–8°C) before laboratory incubation (35–37°C, 48 h) for colony-forming unit (CFU) enumeration. All contact surfaces, including sampling heads, protective covers, and instrument exteriors, were systematically disinfected with 75% ethanol between sampling cycles.

### 2.4 Evaluation criteria

The endoscopy suite is classified as a Class III cleanroom. According to ISO 14644-1 (2015), the particulate concentration under static conditions must comply with ISO Class 7 standards, with maximum permissible limits of 352,000 particles/m^3^ for Dp ≥ 0.5 μm, 83,200 particles/m^3^ for Dp ≥ 1 μm, and 2,930 particles/m^3^ for Dp ≥ 5 μm (28). Under dynamic conditions, the facility must meet ISO Class 8 requirements, corresponding to maximum concentration limits of 3,520,000 particles/m^3^ (Dp ≥ 0.5 μm), 832,000 particles/m^3^ (Dp ≥ 1 μm), and 29,300 particles/m^3^ (Dp ≥ 5 μm). For microbial control, static conditions necessitate adherence to GB 50333–2013 (Architectural Technical Code for Hospital Clean Operating Departments), stipulating that airborne bacterial concentrations remain <150 CFU/m^3^ (27). During operational (dynamic) states, compliance with DB11/T 408–2016 (Specification for Pollution Control in Hospital Clean Operating Departments) is mandatory, requiring airborne bacterial concentrations to be maintained at ≤450 CFU/m^3^ (29).

### 2.5 Statistical analysis

All data were analyzed using Microsoft Excel 2019 (Microsoft Corporation, USA) and SPSS Statistics version 26.0 (IBM Corp., USA). Continuous variables following a normal distribution were expressed as mean ± standard deviation (SD), while non-normally distributed data were reported as median and interquartile range [IQR; M(P25, P75)]. For normally distributed data: Longitudinal comparisons across time points were analyzed using repeated-measures ANOVA. Independent two-group comparisons were conducted with the Student’s *t*-test. For non-normally distributed data: Longitudinal comparisons were evaluated using the Friedman rank-sum test, followed by *post-hoc* Wilcoxon signed-rank tests with Bonferroni correction for pairwise comparisons. Independent two-group comparisons were performed using the Mann–Whitney U test. Multivariate linear regression was employed to identify factors associated with microbial and aerosol concentrations. Variables with *p <* 0.1 in univariate analyzes were included in the final regression model using a stepwise approach. A two-tailed *α* level of 0.05 was adopted, and statistical significance was defined as *p <* 0.05.

## 3 Results

### 3.1 Qualified rates and temporal trends of aerosols by particle size

**Figure 2:**
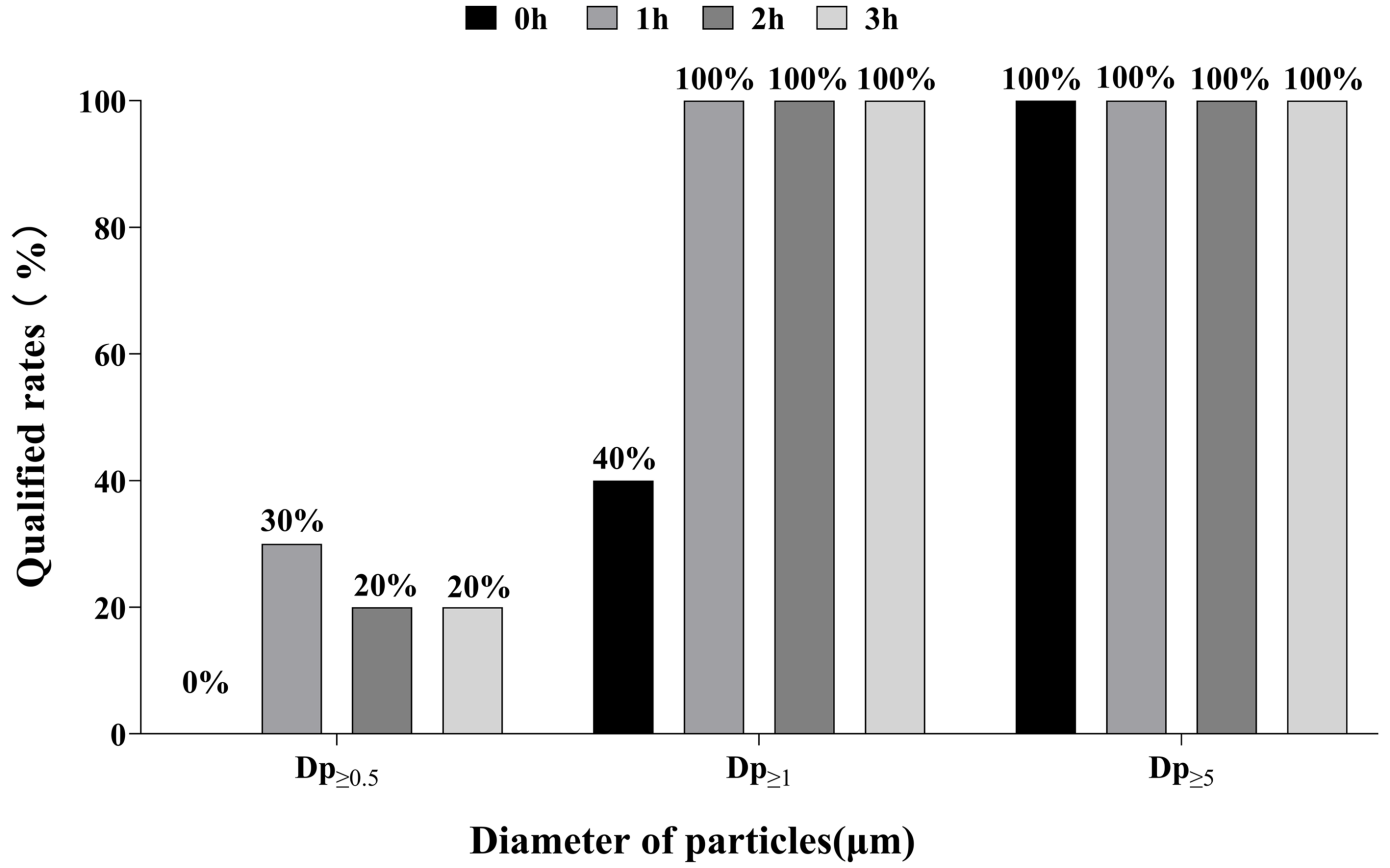
Aerosol qualified rates.

According to ISO standards, Dp_≥0.5_ has a qualified rate of 0 to 30% at 0, 1, 2 and 3 h. In contrast, Dp_≥1_ increased rapidly from 40 to 100% in about 1 h and remained stable thereafter. All Dp_≥5_ maintained 100% qualified rate throughout monitoring (Figure 2).

Aerosol concentrations increased temporally across all particle sizes. Specifically, aerosols with Dp_≥5_, Dp_≥10_, Dp_≥25_, and Dp_≥5–10_ demonstrated statistically significant differences across different periods (*p* ≤ 0.001) (see Supplementary Table 1).

**Figure 3:**
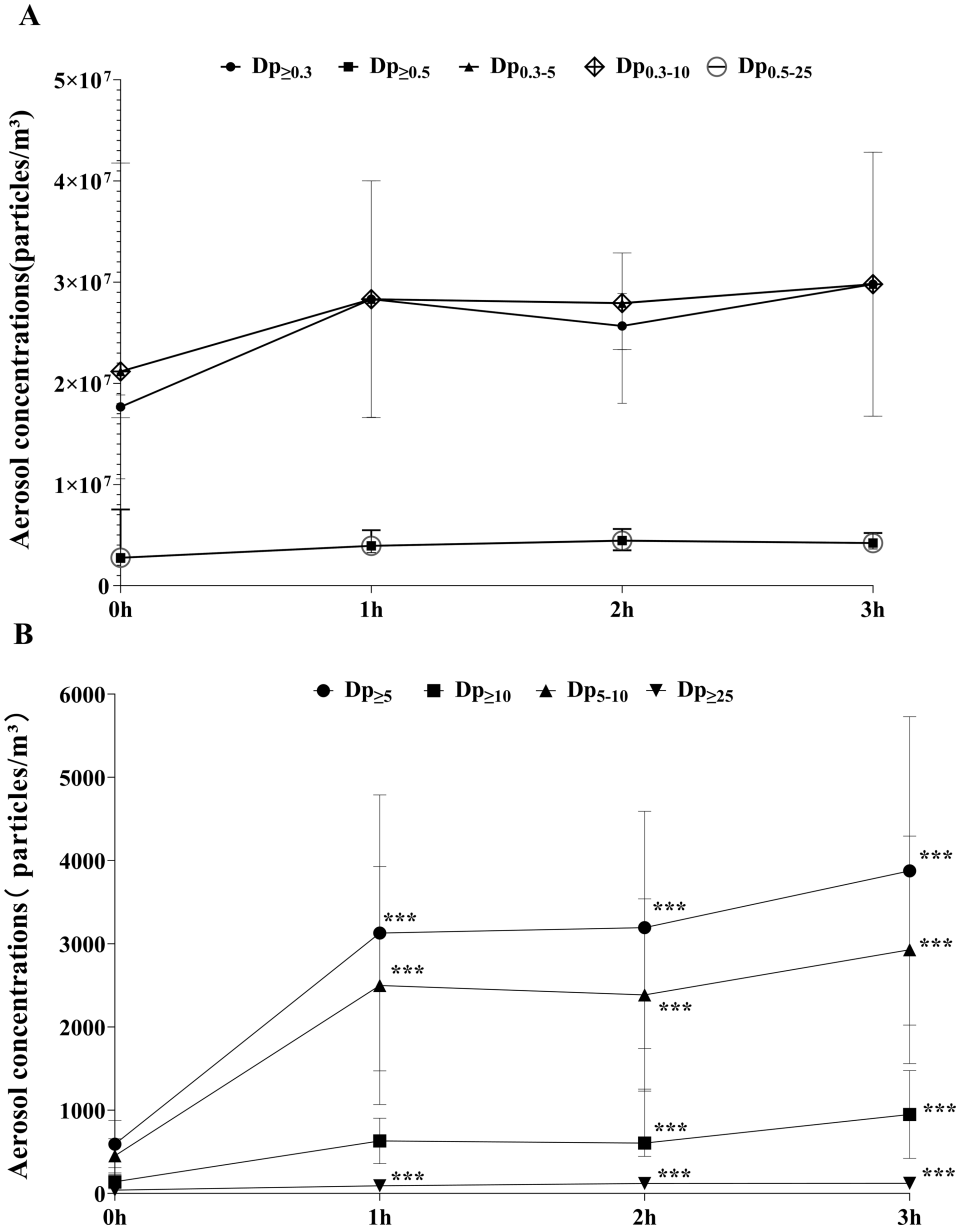
**(A)** Temporal trends of aerosol concentrations for different particle sizes (Dp ≥ 0.3, Dp ≥ 0.5, Dp ≥ 0.3–5, Dp ≥ 0.3–10, Dp ≥ 0.5–25); **(B)** Temporal trends of aerosol concentrations for different particle sizes (Dp ≥ 5, Dp ≥ 10, Dp ≥ 5–10, Dp ≥ 25). Data are presented as mean± standard deviation or M(P25, P75) (see Supplementary Table 1). ***Indicates that the difference was statistically significant compared with 0 h (*P <* 0.05).

Overall, aerosol concentrations showed a relatively consistent pattern: a sharp increase from 0 h to 1 h, stabilization between 1 h and 2 h, and a subsequent peak at 3 h. This trend was more evident in aerosols with Dp_≥5_ (including Dp_≥5_, Dp_≥10_, Dp_5-10_). Notably, the trends for Dp_0.3–10_ and Dp_0.3–5_ were nearly identical, as were the trends for Dp_0.5–25_ and Dp_≥0.5_ (Figures 3A,B). Statistical results revealed that the concentration differences of the particles listed in Figure 3A across various working times were not statistically significant (*p* > 0.05). In Figure 3B, compared to 0 h, Dp_≥10_ increased significantly at 2 h and 3 h (*p* = 0.002, 0.000), while Dp_≥5_, Dp_5-10_, and Dp_≥25_ were significantly higher than baseline levels at 1 h, 2 h, and 3 h (*p* = 0.005, 0.002, 0.002; *p* = 0.008, 0.003, 0.001; *p* = 0.000, 0.000, 0.002). When comparing aerosols concentrations of different particle sizes at other time points, there were no statistically significant differences (*p* > 0.05) (Table 1).

**Table 1:** Pairwise comparisons of aerosol concentrations at different times.

Time		*p*			
		Dp_≥5_	Dp_≥10_	Dp_≥5–10_	Dp_≥25_
0 h	1 h	0.005	0.146	0.008	0.000
	2 h	0.002	0.002	0.003	0.000
	3 h	0.002	0.000	0.001	0.002
1 h	2 h	1.000	0.995	1.000	0.105
	3 h	0.352	0.146	0.962	0.216
2 h	3 h	0.644	1.000	0.998	1.000

### 3.2 Comparative analysis of aerosol growth rate

#### 3.2.1 Dp₀.₃–₅ vs. Dp₅–₁₀

**Figure 4:**
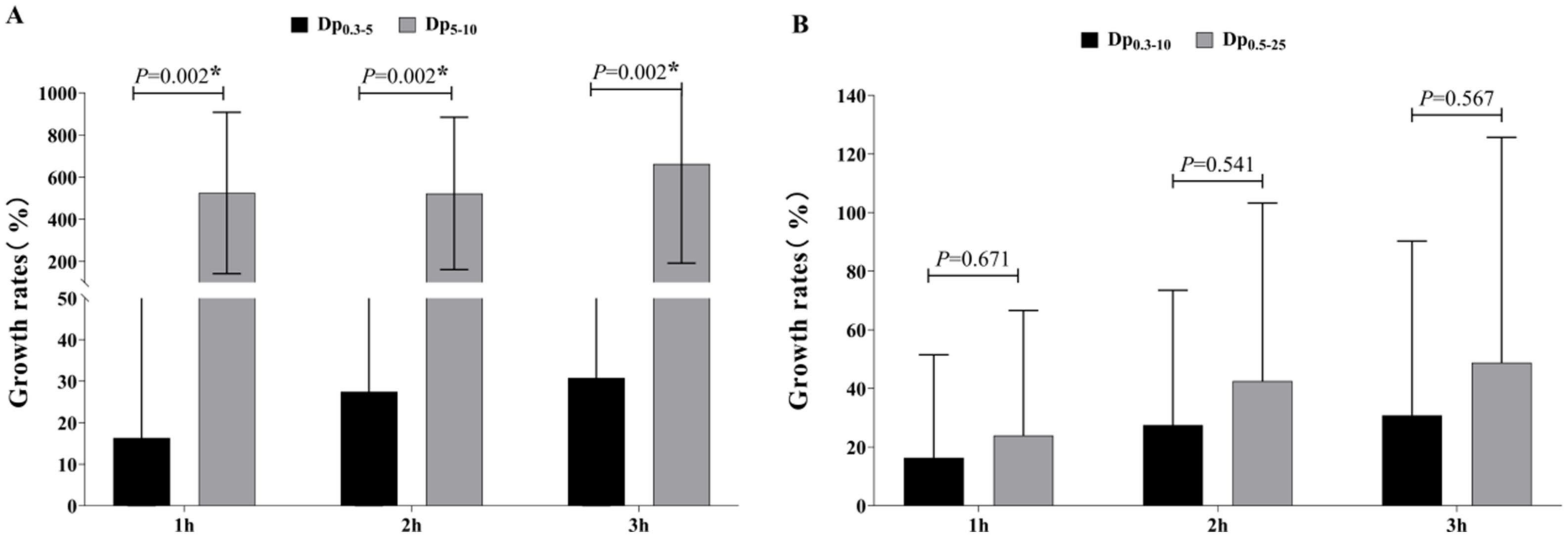
The growth rates of aerosol concentrations compared to the baseline level. **(A)** Comparison of growth rates of Dp_0.3–5_ and Dp_5-10_ at Different Times; **(B)** comparison of growth rates of Dp_0.3–10_ and Dp_0.5–25_ at different times. Data are presented as mean ± standard deviation. *Indicates a statistically significant difference (*p <* 0.05).

Figure 4A present the growth rates of Dp_0.3–5_ and Dp_5-10_ compared to the baseline level. The datas exhibited normal distribution, however, a corrected *t*-test was employed due to non-uniform variances. The results revealed that the growth rate of large-sized particles (Dp_5-10_) was significantly higher than that of small-sized particles (Dp_0.3–5_) (*p <* 0.05). Specifically, Dp_5-10_ showed nearly identical growth rates at 1 h and 2 h, reaching 6.22–6.25 times the baseline level, with the highest growth rate observed at 3 h (7.63 times the baseline). In contrast, the growth rate of Dp_0.3–5_ increased progressively over time, peaking at 1.3 times the baseline level (see Supplementary Table 2 for calculation).

#### 3.2.2 Dp₀.₃–_10_ vs. Dp_0.5–25_

As both datasets exhibited normal distribution and homogeneous variances, a *t-*test was performed. The difference in growth rates for Dp_0.3–10_ compared to Dp_0.5–25_ was not statistically significant at any of the three time points (Figure 4B). However, the growth rate of Dp_0.5–25_ was higher than that of Dp_0.3–10_, with Dp_0.3–10_ growing up to 1.3 times the baseline level and Dp_0.5–25_ growing up to 1.48 times the baseline level (see Supplementary Table 3 for calculation).

### 3.3 Microbial monitoring results

The total number of airborne microbial colonies peaked at 2 h and then decreased slightly. The highest baseline colony count was 10.5 CFU/m^3^, well below the standard limit of 150 CFU/m^3^. All measured values at 1 h (138.5 CFU/m^3^), 2 h (145.5 CFU/m^3^), and 3 h (125.5 CFU/m^3^) remained under 450 CFU/m^3^, with a 100% microbiological qualification rate. Given the non-normal distribution of colony counts (*p <* 0.05), the Friedman rank sum test was performed. Statistically significant differences in colony counts across time points were identified (*χ*^2^ = 18.875, *p <* 0.001) (Table 2). *Post-hoc* Bonferroni analysis demonstrated that colony counts at 1 h, 2 h, and 3 h were significantly elevated compared to baseline (all *p <* 0.05). However, no significant differences were observed between other time points (all *p* > 0.05) (Table 3).

**Table 2:** Microbial monitoring results (Friedman test).

Time	Colony counts [M(P25, P75)/x̄ ±SD, CFU/m^3^]	Qualified rates (%)	Normality test	Friedman test	
			*p*	Χ^2^	*p*
0 h	7.25 (5.88, 9.38)	100%	0.000	18.875	0.000
1 h	34.25 (26.50, 48.00)	100%	0.000		
2 h	62.90 ± 44.98	100%	0.186		
3 h	43.75 (34.13, 83.88)	100%	0.027		

**Table 3:** Bonferroni pairwise comparisons of colony counts.

Time		*p*
0 h	1 h	0.015
	2 h	0.002
	3 h	0.001
1 h	2 h	1.000
	3 h	1.000
2 h	3 h	1.000

### 3.4 Factors associated with aerosol and colony counts

No aerosol-related factors were identified, therefore, only the results of microbial factor analysis were presented. With colony counts as the dependent variable, variables showing *p* ≤ 0.1 in univariate analysis were selected as independent variables. Although the *p*-value for one variable marginally exceeded 0.1 (*p* = 0.103), it was retained given the minimal deviation. Four continuous variables were ultimately included: work duration, polyps detected, biopsy procedures performed, and procedure duration, all entered as raw values (see Supplementary Table 4). Collinearity analysis showed that the variance inflation factor (VIF) was 1.217–1.328 (all<10), indicating no significant multicollinearity. Regression analysis revealed that all four variables significantly affected colony count (*p <* 0.05), collectively accounting for 46.6% of the variance (Table 4).

**Table 4:** Multiple linear regression results for colony counts.

Factors	Estimate	Standard error	Standardized estimate	*t*	*P*	VIF
Constant	62.839	23.904	–	2.629	0.014	
Work duration (h)	22.368	6.868	0.489	3.257	0.003	1.226
Polyps detected (*n*)	13.774	6.206	0.340	2.219	0.036	1.274
Biopsy procedures performed (*n*)	9.151	2.879	0.497	3.179	0.004	1.328
Procedure duration (min)	−3.154	0.775	−0.609	−4.071	0.000	1.217

R^2^ = 0.540, Adjusted R^2^ = 0.466, F = 7.332, *P* = 0.000.

### 3.5 Microbial identification

All blank culture dishes used for control showed no colony growth, and the culture dishes used for sampling presented colonies after cultivation. A total of 16 samples from a particular day were randomly selected during the experiment and subjected to microbiological identification by specialists in the Department of Laboratory Medicine at our hospital. After Gram staining, bacterial samples were observed for cell morphology and staining characteristics using an optical microscope; Fungal samples were preliminarily classified based on colony morphology characteristics, and colonies with unclear features were further identified by microscopic observation to determine their species. A total of 12 microorganisms were detected in the air samples. Bacteria accounted for approximately 79.6% of the total microorganisms, including six Gram-positive species: *Micrococcus*, *Staphylococcus warneri*, *Bacillus megaterium*, *Bacillus licheniformis*, *Microbacterium testaceum* and *Bacillus thuringiensis*; One Gram-negative species: *Pseudomonas luteola*; Fungi constituted 20.4% of the microbial population, comprising *Penicillium*, *Aspergillus niger*, *Aspergillus fumigatus*, *Aspergillus flavus*, and *Rhizopus oryzae* (Table 5).

**Table 5:** Microbial identification.

Type		Pathogen	Number	Proportion
Bacteria	Gram-positive	Micrococcus	51	24.2%
		*Staphylococcus warneri*	40	19.0%
		*Bacillus megaterium*	30	14.2%
		*Bacillus licheniformis*	12	5.7%
		*Microbacterium testaceum*	8	3.8%
		*Bacillus thuringiensis*	8	3.8%
	Gram-negative	*Pseudomonas luteola*	19	9.0%
Fungi		Penicillium	10	4.7%
		*Aspergillus niger*	9	4.3%
		*Aspergillus fumigatus*	8	3.8%
		Aspergillus flavus	8	3.8%
		Rhizopus oryzae	8	3.8%

## 4 Discussion

### 4.1 Qualified rate analysis of aerosols and microorganisms

The endoscopy suite selected in this study is a closed room without windows, which limits natural ventilation. The room relies only on the Ventilation Mechanical Control (VMC) system to complete air exchange. In this study, the qualified rate of aerosols showed different patterns of change in different particle size ranges and time: Dp_≥5_ consistently maintained 100% qualified rate, Dp ≥ 1 fully met the standard within 1 h and remained stable, while Dp _≥ 0.5_ exhibited an initial qualified rate of 0% and improved by only 20–30% after 1–3 h of operation. This result suggested that there was particle size selectivity in the removal efficiency of the VMC for aerosols, which was significant for aerosols above 5 μm, but had relatively limited purification ability for particles ranging from 0.5 to 1 μm. It is noteworthy that the qualified rates of Dp ≥ 0.5 and Dp ≥ 5 after 1 h in this study were significantly higher than the results reported by Gao et al. (30) (both qualified rates were 0%). We considered that this discrepancy mainly stemmed from the following mechanisms: Firstly, 20 cm below the air supply outlet is the direct impact area of the airflow where the velocity of the airflow is high, and aerosols are easily carried and dispersed by the airflow (31), resulting in a higher concentration at the time of the test. This may be a major reason for the failure in Takano’s study. Secondly, the design and operation status of the ventilation system have a significant effect on the aerosol distribution near the air supply outlet. If the efficiency of the ventilation system filters decreases or maintenance is insufficient that may lead to the accumulation of aerosols with Dp ≥ 0.5 and Dp ≥ 5 in this area. Conversely, at a height of 0.8 m above the floor, some of the aerosols had settled on the surface of objects or diffused and diluted within the room (32). Consequently, Dp ≥ 0.5 and Dp ≥ 5 aerosol concentrations were relatively low, and the qualified rate was higher in this study.

This study also revealed that the total colony counts at the four times range from 4.5 to 145.5 CFU/m^3^, which is in accordance with the national standard and consistent with the findings of Jin et al. (33) and Cabo et al. (34). This may be attributed to the standardized use of personal protective equipment (PPE) by HCWs during endoscopic operations and effective cleaning and disinfecting of the environmental surfaces, which make the microbial risk in continuous endoscopic consultation manageable. However, it is specifically noted that the Airborne bacteria sampler used in this study can only detect airborne culturable bacteria and fungi, while other bioaerosol sources such as viruses and parasites cannot be captured (35, 36)^.^ Therefore, achieving microbial concentrations does not equal to fully controllable bioaerosol risk, and relying on microbial detection alone to assess the risk of microbial aerosols may underestimate the risk of exposure for HCWs.

### 4.2 Analysis of aerosol particle size distribution and microbial growth trends

A notable surge in aerosol particle size was observed within the first hour of patient admission, likely attributable to preparatory activities (e.g., instrument arrangement, surface disinfection) that triggered the resuspension of surface-bound aerosols. This phenomenon was further compounded by continuous pollutant release during endoscopic procedures, as supported by Qian et al. (37) We observed a stabilization or slight decrease in aerosol concentration within 1–2 h, which may be associated with the VMC system’s operational efficiency and stabilized human activity (38). However, a slight rebound was observed at 2–3 h, possibly resulting from: (1) cumulative deposition and resuspension of settled particles, (2) prolonged patient presence in densely arranged waiting areas for more than 2 h, and (3) aerosol generation rates exceeding the VMC system’s purification capacity during peak operational phases. Nevertheless, no significant differences were observed between 3 h and earlier time points, indicating the possibility of stochastic variability or uncontrolled confounders. Notably, this interpretation was constrained by a 3-h monitoring window which limited extended temporal inferences.

The total bacterial count exhibited an ascending trend at 0–2 h, likely driven by microbial release associated with endoscopic procedures, staff activities, and the initial activation phase of the VMC system. The peak contamination level observed at 2 h was followed by a marginal decline, which may be attributed to the VMC system’s gradual transition to stable operation (typically requiring 1–2 h post-activation), enabling efficient removal of bioaerosol-bound microorganisms. However, the post-peak reduction showed no significant difference compared to the 2 h maximum (*p* = 1.000), suggesting that these fluctuations might represent inherent stochastic variations within monitoring period.

### 4.3 Comparison of growth rates between Dp_0.3–5_ and Dp_5-10_, Dp_0.3–10_ and Dp_0.5–25_

Aerosols with aerodynamic diameters ≤10 μm can penetrate the respiratory tract. Specifically, particles in the Dp_5–10_ predominantly deposit in the upper respiratory tract (e.g., pharynx and trachea), exerting limited health effects, whereas Dp _<5_ reach the lower respiratory tract (e.g., bronchi and alveoli) and may disseminate systemically via the bloodstream, posing substantial health risks (39). This study stratified aerosols into Dp_0.3–5_ and Dp_5–10_ to compare their growth rates. Notably, Dp_5–10_ exhibited significantly higher growth rates than Dp_0.3–5_, contrasting with Pereira’s observation of predominant Dp_0.3–5_ generation during gastrointestinal endoscopy. This divergence is presumably attributable to methodological distinctions: Pereira et al. (23) strategically positioned sampling probes 1 m above oral/anal orifices to compare aerosol dynamics between two endoscopic procedures, specifically characterizing aerosol generation patterns during gastroscopy and colonoscopy. In this setting, transient events such as coughing, gas expulsion, or scope insertion/ withdrawal, have been demonstrated to dominate aerosol release. In contrast, our study employed uniformly distributed sampling points positioned at a height of 0.8 m to approximate ground-level conditions, enabling a comprehensive assessment of aerosol profiles in clinical environments. This configuration preferentially captures larger particles (Dp_5–10_) due to gravitational sedimentation and airflow-driven aggregation. Furthermore, the movement of transfer carts likely promoted re-entrainment of settled particles, amplifying Dp_5–10_ aerosol concentrations.

Microbial aerosols comprise fungal (0.5–30 μm) and bacterial (0.3–10 μm) components (40, 41). However, as the maximum collection particle size of the instrument employed in this study was 25 μm, the classification ranges were adjusted to Dp_0.5–25_ for fungal and retained at Dp_0.3–10_ for bacterial. Both categories exhibited a 1.16–1.48-fold increase in concentration over 3 h. As Song et al. (42) have proposed potential sources comprising patient secretions, suboptimal endoscope reprocessing, elevated humidity, stagnant water, and insufficient hand hygiene among HCWs. The persistent upward trend in microbial aerosol concentrations underscores the necessity of adaptive air disinfection protocols to mitigate pathogen accumulation during prolonged procedures. However, the observed growth patterns remain inferential and require validation through targeted sampling and culture analysis in the future.

### 4.4 Analysis of influencing factors

Contrary to previous reports by Sagami et al. (15) we excluded patient-related subjective factors such as coughing, vomiting, and flatulence, and we did not consider HCW movements as influencing variables, since these were not restricted in our protocol. Instead, we focused on more objective parameters. Multiple linear regression revealed positive correlations between microbial concentrations and three operational factors: work duration, number of polyps detected, and frequency of biopsy sampling. Interestingly, a negative correlation was observed between microbial levels and procedure duration. Kodikara et al. (43) demonstrated that prolonged procedure time increases microbial contamination through the compounding effects of patient-related factors, staff movement, and equipment relocation. Contrary to expectations, prolonged operating duration exhibited an inverse correlation with microbial contamination levels. We attribute this anomalous phenomenon to four reasons: (1) the regression coefficient of procedure duration (−3.154) was substantially smaller than those of the other three factors, suggesting either minimal actual impact on the dependent variable or insufficient statistical power stemming from limited sample size (44, 45). (2) Potential influence of confounding factors: Variables not fully controlled in this study, such as HCWs seniority and procedural proficiency (more experienced personnel typically perform procedures more efficiently and adhere more strictly to protocols like hand hygiene and surface disinfection, potentially reducing microbial release) and patient health status (e.g., long-term corticosteroid use may cause skin or mucosal dysbiosis, increasing microbial shedding risk), may mechanistically contribute to the observed negative correlation (25, 46, 47). (3) Continuous purification effect of the fresh air system: prolonged operation provides more time for the continuously running fresh air system to dilute and remove microbial aerosols from the air, which could be a key reason for the progressive decline in colony counts observed at environmental sampling points (48). (4) Limitations of traditional microbial culture methods: The monitored colony counts may substantially underestimate the actual abundance and diversity of microorganisms present in the environment.

The biopsy channel is connected to the gastrointestinal tract via a valve that opens temporarily during the insertion or withdrawal of biopsy forceps, thereby releasing airborne microorganisms. Higher biopsy counts have been demonstrated to correlate with increased valve openings and elevated airborne microbial concentrations, which is consistent with the findings of Passi et al. (25) Coughlan et al. (49)hypothesized that polypectomy employing loopers with blunt plastic tips potentially widens valve openings compared to metal forceps, thus possibly amplifying microbial release. Therefore, it was recommended that forceps and loopers be removed from the biopsy channel by wrapping the opening of the biopsy channel with gauze, and that gauze should be promptly placed in a lidded medical waste canister after removal. Meanwhile, endoscopists and nurses should enhance their professionalism to improve the accuracy of biopsy/polypectomy and avoid unnecessary lifting and insertion maneuvers. While Passiet et al. (25) have demonstrated that biopsy procedures elevate microbial loads and aerosol generation, our analysis of Dp_≥0.5_, Dp_≥1_, Dp_≥5_ failed to establish biopsy frequency as a significant determinant. This discrepancy may be attributed to the predominant aerosol size range is 65-145 μm that emitted from biopsy channels, with 100 μm particles requiring only 10 s to settle 3 meters (23, 50). Distant sampling locations likely impaired detection of these rapidly settling aerosols. It may be the most critical reason for failing to confirm the number of biopsies as an influencing factor on aerosol.

### 4.5 Microbial identification results analysis

The most common microbial species in hospital environments include Gram-negative bacteria, Gram-positive bacteria, and fungi. The bacteria identified in this study were classified as opportunistic pathogens, with Gram-positive bacteria constituting the highest proportion, approximately 70%, consistent with findings from a contamination survey of protective garments worn by upper gastrointestinal endoscopists in Japan. Analysis of bacterial sources revealed that 69% originated from oral resident bacteria, while 31% were opportunistic bacteria derived from water used during endoscopic examinations (24). These pathogens can spread via air, water, or contaminated instrument surfaces or persist in the environment due to inadequate disinfection. For instance, spore-forming bacteria such as *Bacillus megaterium* and *Bacillus licheniformis* exhibit strong resistance to disinfectants and high temperatures, complicating their eradication (51, 52). Concurrently, fungi like Aspergillus and Rhizopus thrive in humid environments, and their airborne spores elevate the risk of infection for HCWs and patients. The VMC system may represent a primary transmission route, as moisture in cooling coils promotes microbial proliferation, facilitating dissemination through ventilation ducts (53, 54). Notably, fungal spores can survive on surfaces exposed to patients, HCWs, or air for at least 24 h and often persist for weeks, further amplifying infection risks (55, 56). In hospital settings, airborne microorganisms such as Micrococcus and *Staphylococcus warneri*, while generally low-risk for healthy individuals, may cause infections in immunocompromised hosts. These bacteria are transmitted through contact with contaminated surfaces and form biofilms on medical devices, increasing the risk of cross-infection. *Staphylococcus warneri*, in particular, requires heightened vigilance due to its enhanced pathogenicity and antimicrobial resistance (57). Furthermore, *Pseudomonas luteola* has been demonstrated to potentially contribute to the development of nosocomial pneumonia (58).

A significant limitation of this study is the reliance on traditional culture-based techniques. These methods are inherently limited in their ability to effectively capture low-abundance yet potentially pathogenic airborne microbial communities, resulting in a constrained assessment of microbial diversity, with only 12 species identified. Furthermore, we did not assess potential associations between the identified culturable pathogens and health outcomes among HCWs, such as respiratory infections. Consequently, the true health risk posed by the environmental microbiome to HCWs may have been underestimated. In contrast, studies employing high-throughput sequencing technologies have uncovered more diverse and comprehensive microbial profiles and have successfully established associations between specific microbial communities and respiratory infections. For instance, two studies in Chinese university dormitories and Malaysia schools identified several genera in Gammaproteobacteria (e.g., Haemophilus, Klebsiella, Buttiauxella, and Raoultella) and fungal taxa as being positively associated with respiratory infections (59, 60). Furthermore, *Aspergillus fumigatus*, identified by culture in our study, is prevalent in the sputum of asthma patients yet represents a predominant component of the normal airway mycobiome in this population (61). Critically, previous research demonstrates that the health risks associated with the microbiome are context-dependent rather than inherent properties (60, 62). For example, Šarac et al. (63) reports an inverse correlation between the beta diversity of household bed dust bacteria and childhood asthma risk, but this association is mediated by environmental factors including residential setting, housing type, pet ownership, and cleaning habits. Similarly, classroom exposure to house dust mite allergens and NO2 indirectly affects students’ respiratory health risk through modulating the environmental microbiome(60).

Different from the above architectural environment, the endoscopy room represents a distinct microbial environment. During endoscopic procedures, aerosols potentially containing intestinal microbiota (e.g., *Escherichia coli*, Enterococcus spp.) may be released. The use of chlorinated disinfectants can selectively inhibit certain multidrug-resistant Gram-negative bacteria (e.g., *Pseudomonas aeruginosa*, Acinetobacter spp., and Klebsiella spp.); however, it may also lead to the enrichment of disinfectant-resistant bacteria or fungi (such as Bacillus spp., Aspergillus spp.) in the environment (64, 65). Additionally, patient factors such as immune status and mobility may contribute additional complexity to the environmental microbiome. Consequently, the bacteria and fungi isolated and identified in our study represent only a subset of the microbial communities surviving under these specific environmental selection pressures. A comprehensive understanding of their phylogenetic lineages and potential pathogenicity warrants further investigation. Future research should integrate specific environmental factors of the endoscopy room (e.g., room layout, staff turnover, medical procedures, disinfection protocols, ventilation systems) with advanced technologies like high-throughput sequencing. This integrated approach will elucidate the association between the environmental microbiome composition and HCWs’ health outcomes, enabling a more accurate health risk assessment and informing the development of effective infection prevention and control strategies.

### 4.6 Recommendations

The exposure of HCWs and patients to airborne contamination during endoscopy is an important public hygiene issue. To effectively reduce the potential risk of aerosols and microorganisms to patients and HCWs, we propose the following recommendations: (1) strengthen the skill training of endoscopists and nurses, as experienced endoscopists can indirectly reduce the generation of aerosols by reducing the unnecessary movements of the patient (e.g., coughing, body movement, etc.) (66); (2) HCWs should strictly follow hand hygiene norms and enter the inspection area to regularize the wearing of masks to avoid cross-infection; (3) it has been discovered that SARS-CoV-2 degraded 99.99% after 1 h on cotton because the high porosity of cotton accelerated virus droplet adsorption, drying and degradation. In contrast, viruses were more stable and survived longer on disposable PPE surfaces (67). We suggest exploring alternative PPE materials, such as cotton, which have shown promise in other contexts and may be beneficial for virus degradation, environmentally friendliness, and energy efficiency; (4) provide HCWs with routine health checkups and appropriate vaccinations to enhance their resistance to infectious agents (68); (5) AI-assisted early cancer screening and personalized screening recommendations to reduce the number of unnecessary endoscopies and improve diagnostic accuracy (69, 70); (6) hospitals with larger consultation volume should install more efficient air filtration systems, activate them 1~2 h before work, and regularly maintain and monitor microorganisms on the surface inside the air ducts (71, 72); (7) optimize the diagnosis and treatment process and resource deployment to reduce the unnecessary staying time of patients in the waiting area, thus lowering the risk of aerosols and microorganisms deposited on the surface of clothing arising from prolonged exposure to high-density crowded environments (73); (8) where feasible, installation of external suction devices is strongly recommended to effectively mitigate contaminant dispersion during endoscopic procedures (74); (9) given the substantial microbial contamination observed on air samples, water sources, and instrument surfaces (51). Enhanced environmental surveillance and disinfection efficacy verification are imperative for endoscopy suites to ensure the safety of patients and HCWs; (10) conduct multi-omics profiling of indoor microorganisms and metabolites to identify potential microbial sources of metabolites. This approach should be integrated with specific environmental parameters to achieve a more comprehensive understanding of the associations between the microbiome and health risks within this setting; (11) given that most common ventilation and air conditioning systems operate synchronously, their air filtration capabilities may not be fully optimized throughout the day during the spring and autumn seasons. Therefore, we propose the establishment of a season-specific air quality management system, with particular emphasis on enhancing infection prevention and control strategies during these transitional periods. This approach aims to mitigate exposure risks associated with the elevated incidence of respiratory diseases for both HCWs and patients. Finally, we recommend that endoscopy centers customize their infection control measures based on local conditions to reduce aerosol transmission and microbial contamination, thereby ensuring occupational health for HCWs.

### 4.7 Limitations

Although this study provided some insights, there are some limitations. Firstly, the temporal and spatial asynchrony between aerosol sampling and microbial sampling may lead to bias in the results, and the influence of disturbances in the environment on the results could not be completely excluded. Secondly, we did not evaluate the specific parameters of the VMC system but only clarified that it was a G4 primary filtration system, which may have limited full comprehension of the mechanisms of aerosol and microbial transmission. Additionally, the single-center design, methodological limitations and small sample size may restrict the generalizability of the findings.

## 5 Conclusion

This study first revealed the size-specific growth patterns of aerosol contamination and temporal characteristics of microbial exposure in endoscopy suites, providing critical evidence for optimizing infection prevention strategies. We recommend enhancing ventilation efficiency (e.g., installing air filtration systems with high efficiency) and implementing regular monitoring of environmental microbial aerosol burden in clinical practice. The integration of artificial intelligence technologies to optimize diagnostic workflows could effectively reduce HCWs’ exposure risks. Future research should prioritize multi-center, longitudinal dynamic monitoring. Utilizing high-throughput sequencing technologies will be essential to explore the intrinsic links between the endoscopy unit microbiome and HCWs’ health outcomes, ultimately supporting the development of precise infection risk prediction models that can inform clinical decisions in infection prevention and control.

## Data Availability

The original contributions presented in the study are included in the article/Supplementary material, further inquiries can be directed to the corresponding author.
