# Supplementary material for: Quantifying aerosol and microbial exposure of healthcare workers in endoscopy suites: a time-based study
Source: Front Public Health. 2025 Aug 29;13:1634327. doi: 10.3389/fpubh.2025.1634327 (PMC12406866; doi:10.3389/fpubh.2025.1634327)
Supplement: Supplementary file 1 [file Table_1.DOCX]

| Table 1. Aerosol Detection Results (Friedman Test/Repeated - Measures ANOVA) | | | | | | | | | | |
| --- | --- | --- | --- | --- | --- | --- | --- | --- | --- | --- |
| Time | Aerosol Concentration（M(P25,P75)/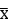±SD, particles/m³） | | | | | | | | | |
|  | Dp_≥0.3_ | Dp_≥0.5_ | Dp_≥1_ | Dp_≥5_ | Dp_≥10_ | Dp_≥25_ | Dp_0.3-5_ | Dp_5-10_ | Dp_0.3-10_ | Dp_0.5-25_ |
| 0h | 17667513.50（10562282.77，18846113.00） | 2768308.00（2298096.72，7526744.66） | 96974.00（63285.34，138900.75） | 592.91±283.42 | 141.19±85.84 | 40.45±18.00 | 21169949.69(17521941.78,41771445.47) | 451.73±204.78 | 21170278.69（16604958.05，41771808.63） | 2768279.03（2298030.80，7526695.25） |
| 1h | 28320091.65±11702317.15 | 3942078.60（3258164.50，5490245.36） | 210779.396±99399.77 | 3129.21±1656.90^*^ | 630.96±273.38 | 92.62±22.08^*^ | 28316962.44±11702510.06 | 2498.26±1430.96^*^ | 28319460.69±11702305.50 | 3941962.65（3258088.94，5490156.55） |
| 2h | 25679877.35（18029632.44，28886378.42） | 4450993.52（3501128.57，5605911.11） | 234675.500±117114.81 | 3193.80±1452.34^*^ | 604.15（444.58，1253.95）^*^ | 119.39±26.08^*^ | 27928929.76(23337920.97，32892364.46 | 2385.02±1155.63^*^ | 27930143.67（23342965.62，32895268.54） | 4450857.59（3501028.74，5605802.21） |
| 3h | 29802002.63±13048649.67 | 4211371.21（3628453.84，5207517.49） | 260854.87(175379.33，280834.25） | 3876.52±1852.53^*^ | 948.63±527.76^*^ | 121.74±35.96^*^ | 29798126.11±13049124.90 | 2927.90±1367.24^*^ | 29801054.00±13048697.69 | 4211230.935（3628308.038，5207403.39） |
| Χ^2^/F | 4.920 | 7.080 | 7.320 | 57.126 | 23.160 | 25.051 | 4.920 | 15.423 | 4.920 | 7.080 |
| P | 0.178 | 0.069 | 0.062 | 0.000 | 0.000 | 0.000 | 0.178 | 0.000 | 0.178 | 0.069 |
| ^*Indicates that the difference is statistically significant compared with 0 h (P < 0.05)^ | | | | | | | | | | |

| Table 2.Comparison of Growth Rates of Dp₀.₃₋₅ and Dp₅₋₁₀ at Different Times( 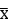±SD, %) | | | | | | | | | | |
| --- | --- | --- | --- | --- | --- | --- | --- | --- | --- | --- |
| Time | Dp_0.3-5_ | Normality Test (P) | Dp_5-10_ | Normality Test (P) | Levene’s test(P) | t’ | P | | | |
| 1h | 16.35±35.14 | 0.513 | 525.18±383.29 | 0.184 | 0.003 | -4.180 | 0.002 | | | |
| 2h | 27.47±46.02 | 0.594 | 522.93±361.65 | 0.455 | 0.002 | -4.298 | 0.002 | | | |
| 3h | 30.80±59.46 | 0.828 | 663.25±471.72 | 0.452 | 0.001 | -4.207 | 0.002 | | | |
| t’: Corrected t-test | | | | | | | |  |  |  |

| **Calculation：**The growth rate calculation follows the formula: [(Time_n_-Time_0_)/Time_0_]×100%. Time0 is the value at 0h, and Time_n_ can be the value at 1h, 2h, or 3h;  Table 3. Comparison of Growth Rates of Dp_0.3-10_ and Dp_0.5-25_at Different Times (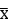±SD, %) | | | | | | | |
| --- | --- | --- | --- | --- | --- | --- | --- |
| Time | Dp_0.3-10_ | Normality Test  (P) | Dp_0.5-25_ | Normality Test  (P) | Levene’s test(P) | t | P |
| 1h | 16.36±35.14 | 0.514 | 23.91±42.69 | 0.221 | 0.427 | -0.432 | 0.671 |
| 2h | 27.49±46.02 | 0.594 | 42.50±60.76 | 0.798 | 0.478 | -0.623 | 0.541 |
| 3h | 30.81±59.47 | 0.829 | 48.77±76.89 | 0.734 | 0.552 | -0.584 | 0.567 |

**Calculation：**The growth rate calculation follows the formula: [(Time_n_-Time_0_)/Time_0_]×100%. Time0

is the value at 0h, and Time_n_ can be the value at 1h, 2h, or 3h;

| Table 4. Univariate Analysis Results of Factors  Univariate Analysis of Factors Associated with Aerosol | | | | | | |
| --- | --- | --- | --- | --- | --- | --- |
|  | PM_≥0.5_ | | PM_≥1_ | | PM_≥5_ | |
| Factors | 95%CI | P | 95%CI | P | 95%CI | P |
| Work duration(h) | -1342738.737-2060612.480 | 0.669 | -34181.236-53938.540 | 0.650 | -594.066-1341.372 | 0.436 |
| Age | -41882.765-263730.731 | 0.148 | -2076.720-6004.474 | 0.328 | -62.934-117.754 | 0.539 |
| Male patients(n) | -619578.639-1128396.233 | 0.556 | -25398.803-20120.042 | 0.814 | -560.642-446.487 | 0.818 |
| Female patients(n) | -769454.028-1037348.026 | 0.764 | -28720.563-17973.436 | 0.641 | -606.240-428.655 | 0.728 |
| HP-positive cases(n) | -2150411.554-313635.654 | 0.705 | -98802.852-36354.932 | 0.352 | -2209.626-777.086 | 0.334 |
| Adenomas detected(n) | -3106069.824-716735.293 | 0.211 | -53907.422-47946.554 | 0.905 | -995.590-1256.202 | 0.814 |
| Polyps detected(n) | -1905889.737-1104223.419 | 0.590 | -31209.074-46938.883 | 0.683 | -658.349-1068.592 | 0.630 |
| Biopsy procedures performed(n) | -669980.584-704836.565 | 0.959 | -9696.226-25395.284 | 0.367 | -218.294-558.559 | 0.377 |
| Gastroscopies(n) | -1245304.438-249169.304 | 0.519 | -67848.185-26155.582 | 0.371 | -1438.408-649.604 | 0.446 |
| Colonoscopies(n) | -617858.816-3416122.881 | 0.166 | -70391.153-37030.159 | 0530 | -1478.990-904.415 | 0.625 |
| Gastroscopy duration (min) | -238958.942-287796.912 | 0.851 | -3900.923-9574.700 | 0.396 | -102.878-196.993 | 0.526 |
| Colonoscopy duration(min) | -262556.910-114123.654 | 0.426 | -5584.355-4272.676 | 0.787 | -141.345-75.536 | 0.539 |
| Procedure duration(min) | -255072.703-128492.861 | 0.505 | -4145.947-5848.611 | 0.730 | -119.345-102.193 | 0.875 |
| BBPS | -956518.597-2360727.041 | 0.393 | -66671.832-18370.126 | 0.254 | -1563.306-300.049 | 0.176 |
| Hp: Helicobacter pylori; n: Number of cases; * Represents meaningful variables; BBPS: Boston Bowel Preparedness Score | | | | | | |

| Univariate Analysis of Factors Associated with Colony Counts | | |
| --- | --- | --- |
| Factors | 95%CI | P |
| Work duration(h) | 1.942-39.396 | 0.032* |
| Age | -1.797-1.492 | 0.851 |
| Male patients(n) | -10.454-7.753 | 0.763 |
| Female patients(n) | -9.462-9.302 | 0.986 |
| HP-positive cases(n) | -23.046-31.805 | 0.746 |
| Adenomas detected(n) | -11.901-28.375 | 0.409 |
| Polyps detected(n) | -2.646-27.248 | 0.103* |
| Biopsy procedures performed(n) | -0.406-12.991 | 0.065* |
| Gastroscopies(n) | -29.660-7.544 | 0.233 |
| Colonoscopies(n) | -15.332-27.708 | 0.561 |
| Gastroscopy duration (min) | -2.716-1.190 | 0.430 |
| Colonoscopy duration(min) | -4.192-1.145 | 0.252 |
| Procedure duration(min) | -3.512-0.300 | 0.095* |
| BBPS | -22.854-11.31 | 0.515 |
| Hp: Helicobacter pylori; n: Number of cases; * Represents meaningful variables; BBPS: Boston Bowel Preparedness Score | | |
